# Enhanced surveillance of hospitalized COVID-19 patients in Europe: an evaluation of the I-MOVE-COVID-19 surveillance network

**DOI:** 10.1093/eurpub/ckad185

**Published:** 2023-10-27

**Authors:** Johanna J Young, Heather Mutch, Angela M C Rose, Josie M M Evans, the European COVID-19 Hospital Evaluation Writing Group, Jim McMenamin, Josie Murray, Ausenda Machado, Ana Paula Rodrigues, Irina Kislaya, Giedre Gefenaite, Monika Kuliese, Ligita Jancoriene, Ieva Kubiliute, Indrė Jonikaitė, Roberta Vaikutytė, Birute Zablockiene, Aukse Mickiene, Amparo Larrauri, Clara Mazagatos, Thomas Demuyser, Els Van Nedervelde, Lucie Seyler, Mihaela Lazar, Florence Galtier, Philippe Vanhems

**Affiliations:** Clinical and Protecting Health Directorate, Public Health Scotland, Glasgow, UK; Clinical and Protecting Health Directorate, Public Health Scotland, Glasgow, UK; Epiconcept, Paris, France; Clinical and Protecting Health Directorate, Public Health Scotland, Glasgow, UK

## Abstract

**Background:**

A pre-existing, well-established European influenza surveillance network known as I-MOVE enabled the rapid implementation of a European multi-country COVID-19 hospital surveillance network for surveillance of hospitalized COVID-19 cases in early 2020. This network included 257 hospitals in 11 surveillance sites across nine countries. We aimed to identify whether the surveillance objectives were relevant to public health actions, whether the surveillance system met its objectives, where and how shortcomings could be improved, and whether the system was sustainable.

**Methods:**

We identified six key attributes (meeting objectives, usefulness, timeliness, data quality, simplicity and sustainability) to assess, using Centers for Disease Control and Prevention’s evaluation framework. We analyzed pooled datasets, held interviews and group discussions with 10 participating and coordinating sites and gathered feedback through web surveys.

**Results:**

There was overall agreement that the surveillance objectives had been met and being involved in a network of European partners had additional important benefits for stakeholders. While the publication of the outputs was not always sufficiently timely, data submission processes were considered straightforward and the key surveillance variables (age, sex, hospital admission and mortality data) were complete. The main challenges were identified as the collection of the large number of variables, limited available human resources and information governance and data protection laws.

**Conclusions:**

I-MOVE-COVID-19 delivered relevant and accurate data supporting the development and implementation of COVID-19 surveillance. Recommendations presented here identify learning opportunities to support preparedness and surveillance response for future pandemics. The applied evaluation framework in this study can be adapted for other European surveillance system evaluations.

## Introduction

In response to the emergence of severe acute respiratory syndrome coronavirus 2 (SARS-CoV-2), many European countries established comprehensive multi-level surveillance systems for COVID-19 cases, to provide critical information for public health decision-making.^1^

The I-MOVE-COVID-19 hospital surveillance network was established by the I-MOVE-COVID-19 Consortium (founded in February 2020 and coordinated by Epiconcept^2^^,^^3^). This was an expansion of the multi-country Influenza-Monitoring Vaccine Effectiveness (I-MOVE) network founded in 2007 to measure influenza vaccine effectiveness (VE) in Europe.^4^ Having this pre-existing, well-established European platform facilitated the development and implementation of COVID-19 hospital surveillance across surveillance sites, as they were able to build on existing common protocols, expand existing information governance procedures and re-train staff already familiar with the surveillance activities.

The surveillance objectives were to (i) describe clinical and epidemiological characteristics of hospitalized COVID-19 SARI patients, (ii) describe the virological characteristics of SARS-CoV-2 in these patients, (iii) improve understanding of severe disease progression to guide patient management and public health response, (iv) strengthen COVID-19 preparedness through hospital surveillance, (v) describe severe COVID-19 cases by sex, age group and risk/protective factors; and (vi) describe in-hospital COVID-19 deaths.^5^ These objectives were established in the early days of the COVID-19 pandemic in a rapidly changing context with much uncertainty as to how the situation would develop. It was envisaged that pooling data from each country, thereby providing a greater sample size, would allow for a more accurate and representative description of hospitalized COVID-19 cases. Surveillance bulletins were prepared quarterly, and regular network meetings were held to share experiences on the implementation processes and developments across each participating site’s surveillance systems.

Any surveillance system, and particularly new systems, should be evaluated to identify possible improvements in performance and the overall public health response. Our evaluation objectives were (i) to identify whether the surveillance objectives were relevant to public health action, (ii) to assess the surveillance objectives and areas for improvement, (iii) to evaluate the sustainability of the surveillance system and finally, (iv) to consider more efficient or alternative routes to achieve the surveillance objectives. We report the results of this comprehensive evaluation of the European COVID-19 hospital surveillance system and provide recommendations to improve the current system and to support the planning and implementation of future enhanced surveillance activities.

## Methods

### COVID-19 hospital surveillance network

**Figure 1:**
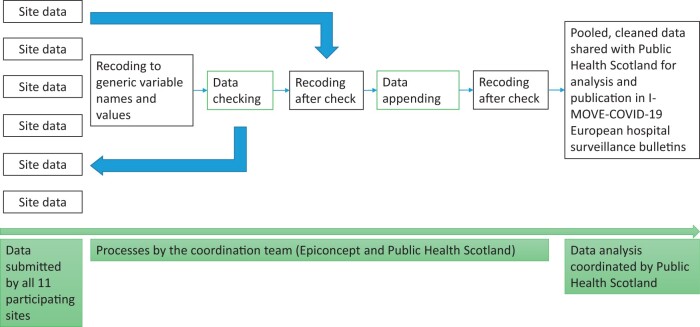
The process for cleaning, pooling and analysing data from 11 sites

Results were collected from 11 surveillance sites, i.e. groups of hospitals and public health institutes within specific European regions that submitted the surveillance data collectively. A total of 257 participating hospitals across nine European countries were included in I-MOVE-COVID-19 (Albania, Belgium, England, France, Lithuania, Portugal, Romania, Scotland and Spain). Spain and France had two participating sites in different regions. With the exceptions of hospitals in England and Scotland—where surveillance was nationwide and register-based via data linkage of routine hospital and infection datasets—an average of three hospitals were included per site. All 23 sentinel hospitals collected COVID-19 hospital data through questionnaire-based surveillance, either on paper or electronically. Every quarter, the participating sites securely submitted their case-based surveillance data to Epiconcept. There were 105 separate variables requested for data submission covering patient demographics, hospital records, severity indexes, risk factors, SARS-CoV-2 presenting symptoms, laboratory test results and clinical information. The data were cleaned and pooled before sharing with Public Health Scotland (PHS), who together with Epiconcept led on analysis (figure 1). Analytical results were presented in quarterly surveillance bulletins that were published on the I-MOVE-COVID-19 website^3^ and used to improve understanding of I-MOVE’s objectives. No ethical approval was required for this evaluation as existing non-identifiable data were used.

### Evaluation framework

The framework for this evaluation was adapted from the ‘Updated Guidelines for Evaluating Public Health Surveillance Systems’ of the Centers for Disease Control and Prevention (CDC).^6^ The surveillance system was assessed against six attributes tailored to suit the scope and the specific evaluation objectives including the achievement of surveillance objectives, usefulness, timeliness, data quality, simplicity and sustainability. Indicators to measure these attributes were defined using adapted CDC and European Centre for Disease Prevention and Control (ECDC) guidelines^6^^,7^ (table 1).

**Table 1:** Methods to evaluate the surveillance system attributes and its set key indicators

Attribute	Indicator	Type of data	Data collection method(s)
**Attribute 1:** The achievement of surveillance objectives	Number, percentage of participating site representatives who think written objectives have been met by the system	Quantitative and qualitative	Web survey, semi-structured interviews/group discussions, document review
**Attribute 2:** Usefulness	**Data collection** Number and list of variables reported which are required but considered not useful at the European level	Quantitative and qualitative	Web survey, semi-structured interviews/group discussions
	Number and list of additional variables not being collected but which would be useful to be collected at European level		
	**Use of European level data for decision-making processes** Use of European level data for decision-making or to improve surveillance (e.g. has this European level data been used to guide policy at national level)	Qualitative	Web survey, semi-structured interviews/group discussions
	**Added value of participation in a European surveillance network** Usefulness of being part of European COVID-19 hospital surveillance network (e.g. added value of European network, networking meetings and surveillance bulletins)	Qualitative	Web survey, semi-structured interviews/group discussions
**Attribute 3:** Timeliness	**Number of days between key steps** Number of days between the dataset received by PHS from Epiconcept completed for analysis and publication date	Quantitative	Document review, analysis of final dataset
	Median number of days between mean admission date across all sites and date of each surveillance bulletin publication		
	Median number of days that data was submitted across all sites prior to agreed submission deadline		
	**Balance between timeliness and information needed** Timeliness and frequency of system reporting (data dissemination) to meet surveillance objectives	Qualitative	Web survey, semi-structured interviews/group discussions
**Attribute 4:** Data quality	Variable completeness	Quantitative	Analysis of final dataset
	Number of excluded cases that did not meet the case definition		
	Stakeholder opinion of data quality (whether they feel it is sufficient to meet objectives)	Qualitative	Web survey, semi-structured interviews/group discussions
**Attribute 5:** Simplicity	**Opinion on simplicity of the surveillance system** Opinion on the data collection process	Qualitative	Semi-structured interviews/group discussions
	Opinion on the data collation	Qualitative	Semi-structured interviews/group discussions
	**Data submission** Opinion on the data collation Person-days for data preparation—need of additional resources (on top of usual workload) done by routine services.	Qualitative and quantitative	Web survey, semi-structured interviews/group discussions
	Feasibility of reporting deadlines for data collection	Qualitative	Semi-structured interviews/group discussions
**Attribute 6:** Sustainability	Plans for continuation of data collection and/or expansion	Qualitative	Semi-structured interviews/group discussions

### Data collection

The evaluation was performed unblinded by three internal evaluators from PHS, part of the I-MOVE-COVID-19 hospital surveillance network, and data were collected through a mixed-method approach using quantitative and qualitative data collection methods, including document review and stakeholder consultation (survey and semi-structured group discussions and interviews). Table 1 shows the data collected for each indicator, reviewed between October and December 2021.

### Document review

All documents and systems related to the surveillance system were identified and reviewed. These included the protocol for I-MOVE-COVID-19 hospital phased surveillance, documents describing standard operating procedures, surveillance bulletins, scientific posters presented at scientific conferences, network meeting agendas and meeting minutes.

### Survey

The indicators of each attribute were used to formulate survey questions that were administered online via LimeSurvey.^8^ The survey was piloted and adapted accordingly prior to its administration. The survey link was sent to all network members from the participating sites. Respondents were asked to identify their role in the surveillance system but otherwise remained anonymous.

### Group discussions/interview

Depending on participant availability, either semi-structured interviews or group discussions were held with the sites, to allow a blend of closed- and open-ended questions, accompanied by follow-up questions. The questions were prepared in advance using the attributes and indicators under surveillance and the key discussion points were shared with the interviewees in advance of the meeting. The sessions were held virtually by the authors using Microsoft Teams between November 2021 and January 2022. Interview notes were recorded on a structured interview template according to each attribute and coded into the most commonly mentioned themes within each indicator by two researchers.

### Analysis of the datasets

Pooled datasets from six data collections with data up to 31 December 2021 were used to derive quantitative indicators (table 1). Timeliness of the surveillance system was measured by calculating the number of days between key steps in the surveillance process; specifically, the time between the initial date of analysis and the median date of admission, and the publication date of the surveillance bulletins. The data quality of the surveillance system was measured by calculating the level of completeness of the collected variables, the proportion of reported cases that fit the case definition^9^ and the number of unanalyzed variables. All analyses were performed using Microsoft Excel.

## Results

### Document review

The I-MOVE-COVID-19 surveillance protocol and the I-MOVE-COVID-19 hospital surveillance evaluation protocol were identified and reviewed, as well as all six pooled datasets submitted during the data collection points, six I-MOVE-COVID-19 surveillance bulletins and two scientific poster presentations presented at The European Scientific Conference on Applied Infectious Disease Epidemiology (ESCAIDE) 2021 conference.^5^^,^^10–16^ All network meeting agendas and meeting minutes of the hospital surveillance system were identified and included in the document review.

### Semi-structured group discussions and interviews and web survey

There were two interviews with individuals from two sites and four group discussions. Overall, all countries and 9 out of 11 participating sites were represented. The link to the web survey was sent to all stakeholders in the network and ten completed surveys were received. The roles of the respondents were varied (diverse and multiple roles could be selected): regional and national public health institute-based network members (*n* = 3), co-ordinators (*n* = 3), data managers (*n* = 2), hospital-based network members (*n* = 2), university-based network members (*n* = 2) and laboratory expert (*n* = 1).

### Evaluation of system attributes

#### The achievement of surveillance objectives

Respondents generally indicated that the surveillance system met all six of its objectives. From the 10 completed surveys received, 8 responded to six parts of this question. Most questions (42/48) were answered by the respondents in (strong) agreement that the surveillance system met its objectives. Five responses were neutral, and only one answer disagreed that the surveillance system met all its objectives.

### Usefulness

#### Data collection

Forty-two percent (61/145) of the requested variables as defined in the study protocol and collected for surveillance purpose (table 3) were selected by at least one respondent as unnecessary for surveillance purposes. They also commented on variables which could be omitted for other reasons, such as postcode which could lead to patient identification, clinical characteristics that did not align with typical epidemiological surveillance, and hospital ward and patient test/scan results as these were free text complicating comparability.

**Table 3:** Average completion rates across all participating sites of the I-MOVE COVID-19 hospital surveillance network and data submissions of variables analyzed and included in the surveillance bulletins, by category

Category	Variable	Definition	Average variable completion rates across all sites and data submissions (%)
Patient characteristics	Age	Age of patient	100
	Sex	Sex of patient	100
	Smoking	Never, former (stopped smoking at least 1 year before inclusion in the study), current smoker	9
	Pregnant	Whether patient is pregnant	24
	Residence	Patient residence at time of SARI onset. Whether patient was living at home or was institutionalized, or had pre-hospital dependence on home support/care	68
	Hcw	Whether the patient is a healthcare worker	48
Hospital information	Admitdate	The hospital admission date of each patient	100
	Icuadmitdate	Date first admitted ICU/HDU	100
	Swabdate	Respiratory specimen collection date	94
	Dischargedate	Date of hospital discharge	63
	Icu	Admission to ICU or HDU	96
	Icudischargedate	Date last discharged from ICU/HDU	96
Severity indicators	Vent	Patient’s level of mechanical ventilation. Note that option 1 is for respiratory support level ECMO, option 2 includes any high-flow (6 l/min or higher, including OptiFlow), and option 3 includes any noninvasive, positive pressure ventilator	84
	Outcome	Indicate the outcome of the patient known at the time of data collection	94
	Deathdate	Date of death	100
	Deathcause	Cause of death	100
Symptoms at admission	Onsetdate	Date of onset of symptoms	65
	Malaise	Malaise	8
	Headache	Headache	9
	Cough	Cough	10
	Sob	Shortness of breath	10
	Sorethroat	Sore throat	8
	Myalgia	Myalgia	9
	Vomit	Vomiting	8
	Diarr	Diarrhoea	9
	Abdopain	Abdominal pain	9
	General_deter	Deterioration of general condition (asthenia or loss of weight or anorexia or confusion or dizziness)	4
	Suddenonset	Sudden onset	2
	Ageusia	Loss of sense of taste	6
	Anosmia	Loss of sense of smell	6
	Dysgeusia	Distortion of the sense of taste	1
	Fever	History of fever	9
	Feverish	Sub-febrility (37–38°C)	2
	Chills	‘Chills’, shivering	2
	Coryza	Coryza	4
	Dizzy	Dizziness	3
	Tach	Tachypnoea or signs of low oxygen saturation	4
	Palp	Palpitations	2
	Nausea	Nausea	3
	Nausea_vomit	Nausea and vomit	3
	Conjunct	Conjunctivitis	7
	Dermato	Rash or other dermatological manifestations of COVID-19	8
	Confusion	Confusion	9
	Chest	Chest pain	6
Underlying chronic conditions	Liverdis	Chronic liver disease (excluding cancer)	69
	Diabetes	Dementia	70
	Heartdis	Heart/cardiac disease (excluding hypertension)	72
	Cancer	Cancer (any)	49
	immuno	HIV (including other immunodeficiency, organ transplantation)	49
	Lungdis	Lung disease (excluding asthma)	66
	Rendis	Renal disease (excluding cancer and acute renal failure)	72
	Dement	Dementia	48
	Stroke	Stroke	12
	Rheumat	Rheumatologic disease	49
	Anaemia	Anaemia/chronic haematologic disease	12
	Tuberc	Tuberculosis	6
	Asplenia	Asplenia (absence of/damage to spleen)	37
	Asthma	Asthma	71
	Hypert	Hypertension	58
	Neuromusc	Neuromuscular disorder	61
	Obese	Obesity (only if height, weight and BMI not collected; can be calculated)	60

Notes: BMI, body mass index; ECMO, extracorporeal membrane oxygenation; HDU, high-dependency unit; ICU, intensive care unit.

Six out of nine interviewed sites reported that too many variables were required, resulting in high levels of missing data for some variables that were not considered core. It was suggested that the number of variables should be determined according to variable completeness levels, and from both the interviewees and respondents, a common view was that if timely data collection is desired then only essential variables, such as patient demographics, hospital information (e.g. hospital admission and discharge dates)and severity indicators should be collected (table 3).

#### Added value of participation in a European surveillance network

Stakeholders identified that a key strength of this surveillance system was that it involved a network of European partners. Having a network supported the understanding of developments across different sites, strengthened relationships with stakeholders, supported advocacy for national COVID-19 hospital surveillance and helped to attract funding from other sponsors to support the surveillance activities. Collaboration within the network allowed sites to automate data flow processes which was recognized and highly praised at the national governmental level.

The quarterly publication of the surveillance data and network meetings were generally perceived positively. This allowed sites to raise awareness of the importance of collecting this data, thereby supporting the implementation and development of their surveillance.

### Timeliness

#### Number of days between key steps in the surveillance process

An average of 39 days (range: 15–63 days) was recorded between the pooled dataset being sent for analysis and publication date (table 2). Across surveillance bulletins, the median number of days between mean admission date and publication was 132 days (table 2). On average, the median date of dataset submission was within 1 day of the agreed submission deadline (table 2).

**Table 2:** Number of days between key steps to assess timeliness in the surveillance system, by surveillance bulletin, I-MOVE-COVID-19 hospital surveillance network, Europe, 2020–21

	Number of days between the dataset received by PHS from Epiconcept and publication date	Median number of days between mean admission date across all sites and date of each surveillance bulletin publication (IQR)	Median number of days that data were submitted across all sites prior to agreed submission deadline (IQR)
Surveillance bulletin 1	15	160 (141–169)	–
Surveillance bulletin 2	51	98 (143–293)	10 (6 to 25)
Surveillance bulletin 3	38	106 (139–345)	5 (−4 to 15)
Surveillance bulletin 4	41	160 (204–458)	0 (−8 to 1)
Surveillance bulletin 5	63	111 (248–362)	−11 (−3 to 13)
Surveillance bulletin 6	26	158 (180–475)	0 (−4 to 1)
Overall (mean)	39	132 (176–350)	1 (−4 to 8)

Notes: No formal deadline was given for the first data submission; therefore, the number of days that data were submitted prior to the agreed submission deadline could not be calculated for surveillance bulletin 1. Minus figures refers to days submitted after the agreed submission deadline.

#### Timeliness and frequency of system reporting (data dissemination) to meet surveillance objectives

Half of the respondents considered the frequency of the publications sufficient to meet the surveillance objectives, although it was suggested that more regular, short, and rapid communications could have been combined with lengthier in-depth surveillance bulletins published less frequently. However, it was also acknowledged that more frequent data submissions would have caused an increased burden on an already-strained workforce.

### Data quality

Key surveillance variables (age, sex, hospital admission and mortality data) had completion rates of 100%. Patient’s type of residence, healthcare worker status, symptom onset date and most chronic conditions had completion rates between 30% and 69%, whereas patient’s smoking status, pregnancy status, three chronic conditions (anaemia, stroke and tuberculosis), and all symptom data had completion rates <25% (table 3). The overall completion rate of the variables varied by site and decreased over time. Data from England and Scotland were the main contributors to this decrease as they supplied more data and their data completion for non-essential surveillance variables decreased over time. An average of 14% (*n* = 9611/69 734) of cases that did not meet the case definition were excluded. The proportion of analyzed variables in the surveillance bulletins varied between 43% and 63%.

#### Stakeholder opinion of data quality

While most interviewees performed regular data management activities to ensure better data quality, such as variable recoding, de-duplication, cross-validation and translation to English at a national level, several sites indicated that these were completed by the hospital sites directly. Generally, interviewees indicated that data quality improved over time. One interviewee suggested introducing conditions and rules during automated data collection to facilitate high data quality and improved completeness levels.

### Simplicity

#### Data collection

Several challenges were identified by respondents around the simplicity of data collection, including information governance processes and following data protection laws, obtaining clearance from data protection bodies or patients, and developing automated data collection approaches. Respondents and interviewees suggested that more frequent updates of the surveillance protocols and training sessions for new staff would be helpful.

#### Data collation

The collation of data was deemed straightforward, although respondents expressed challenges such as the lack of automation in some sites, high workload and the large number of requested clinical variables (e.g. chest X-ray findings, ECG findings).

#### Data submission

Preparing the data for the submission process was considered straightforward by respondents and interviewees. This process took between 2 and 30 days with an average of 11.5 days. Four out of nine sites reported during the interviews that data submission deadlines were challenging due to competing deadlines, lack of human resources, compliance with information governance procedures or delayed data collection in hospitals.

### Sustainability

Sites’ stances differed regarding the continuation of COVID-19 surveillance post the I-MOVE-COVID-19 project. Sites intending to continue their COVID-19 hospital surveillance will do so either through their national surveillance programme or through other integrated European respiratory surveillance networks such as the ‘Vaccine Effectiveness, Burden and Impact Studies’ (VEBIS),^17^ which also includes E-SARI-NET, the European SARI surveillance network. For sites not part of these networks, continuity of the surveillance will depend on the development of the pandemic.

## Discussion

This evaluation of the multi-country European hospital surveillance system for COVID-19, focused on the description of the system and assessment of the surveillance attributes. Survey respondents were in overall agreement that the surveillance system met its objectives.

### Usefulness

This rapidly deployed European surveillance network was an important enhancement to existing surveillance mechanisms used in Europe. Sharing and pooling of European data allowed questions to be answered that individual countries could not answer efficiently alone, particularly where data is scarce (e.g. information on chronic conditions).^18^ Being involved in a European network was also considered extremely valuable, as it assisted individual sites to strengthen national surveillance and supported relationship building with different national and international stakeholders. Cross-country cooperation and data sharing have been recognized to support the centralization of efforts, disseminate information and better prepare and respond to global health challenges.^19^^,^^20^ Combining the expertise and resources of I-MOVE influenza, an existing European multidisciplinary network, to include COVID-19 has shown that European surveillance systems can be adapted in a timely and flexible way suggesting potential for adaptability to also include other pathogens. While the experience and infrastructure from this surveillance network could form a foundation for future emerging pathogens, it is acknowledged that the adaptability of the surveillance system would still depend on various factors such as the nature of the pathogen, the availability of data, and coordination between different stakeholders.

Many hospitals and clinical staff were overburdened during the pandemic, so the collection of the large number of variables required at the start of the pandemic to better understand this novel virus in a hospital setting was challenging. Data collection should be designed to meet the information needs of public health decision-makers, the public and health workers.^21^ It is key that the benefits of collecting additional variables are balanced against the potentially increased burden on clinical staff. A minimum dataset for integrated influenza/SARS-CoV-2 sentinel surveillance is recommended to support the data quality and sustainability of future surveillance systems.^21^^,^^22^

### Timeliness

The publication of the bulletins was not timely enough to influence public health actions. However, most sites shared their own data prior to publication to inform decision-makers and ensure an adequate response. It is acknowledged that improved timeliness of the reporting of pooled and therefore more powerful analyses could have influenced wider public health actions but it was also important not to compromise the validity and quality of the data, and to burden sites with additional data collection and submissions. The development of automated data submission systems linked to electronic databases would address this problem, but the development of such systems across Europe is heterogeneous.^23^

### Data quality

Basic demographic data generally had high completeness levels, whereas more specific patient characteristics were less complete. While some issues stemmed from clinicians being overwhelmed by the pressures of the pandemic, other reasons for incomplete data collection included difficulty obtaining ethical permission for certain variables, patients’ unwillingness to disclose sensitive information^21^ and symptom data not always being routinely reported in hospital settings. While sites that collected data through a sentinel questionnaire-based approach generally reached higher levels of data completeness, sites that collected their data through linkages of national registers accrued more cases. These sites supplying more data submitted fewer non-essential surveillance variables, resulting in lower variable completeness. The World Health Organization (WHO) suggests that good quality data with timely reporting, even from fewer sites, are more useful than a large volume of poor-quality data not reported in a timely manner.^22^ It is therefore key that sites collecting data on more cases assess whether they can be effectively managed, monitored and sustained, while considering the increased human and financial resources, technical and operational assistance that may be required.^22^

### Simplicity

While the operation of the COVID-19 hospital surveillance was generally perceived as straightforward, the main challenges were associated with data collection processes. The collection of COVID-19-specific hospital data has been previously identified as an additional burden for clinical staff.^24^ To facilitate the collation and analysis of data at the national level, countries may consider establishing or strengthening electronic data platforms that link epidemiological and virologic data to sentinel and non-sentinel surveillance systems and are accessible to stakeholders.^22^ Flexible electronic systems should support data collection and transfer in the changing COVID-19 pandemic situations as well as in other emerging outbreaks.^25^

### Sustainability

The sites took different positions on the continuation of COVID-19 hospital surveillance, with some planning to continue and others depending on the pandemic’s development. ECDC and WHO recommend integrated respiratory surveillance of COVID-19, influenza and other respiratory pathogens.^21^^,^^22^ While most European countries have established comprehensive surveillance systems for COVID-19 with a large proportion reporting all positive cases regardless of indication for testing, European countries are now being encouraged to transition from emergency surveillance to more sustainable and objective-driven surveillance systems.^21^ As a result, integrated European respiratory surveillance networks that include COVID-19 hospital surveillance have been developed.^17^

### Limitations

This evaluation has some limitations. The variance of the different types of surveillance systems (sentinel vs. national surveillance), as well as the discrepancies in datasets, number of collected variables and completion rates, made comparisons challenging. The evaluation was performed during an emergency response, which may have reduced stakeholder participation, thereby reducing representativeness from participating sites. For example, due to staffing and resource issues, it was not possible to arrange separate one-to-one interviews with all stakeholders from all participating sites which would have provided the most comprehensive picture, but we were able to arrange either group discussions or interviews with representatives from all countries and nine out of 11 sites. In contrast, the response rate of the survey was low, despite reminder e-mails being sent to the network in attempts to improve the response rate. Responses to certain questions of the survey may also have reflected the respondent’s perception of the current system within their site or specific area of work and may not necessarily provide a general view of the overall participating site. Although facilitators attempted to mitigate dominance bias, responses may have been biased towards participants with strong opinions. The evaluation was performed by internal evaluators, which enabled a strong understanding of the system, an extended period of participant observation, and the ability to make ongoing improvements throughout the implementation. However, this may have introduced confirmation bias and inhibited their ability to act purely as objective outsiders.

### Recommendations

Our evaluation has generated the following recommendations specific to any future deployment of an integrated respiratory surveillance system:

#### Surveillance methods

- Increase the timeliness of reporting data to support public health actions in a timely fashion.
- Reduce the minimum mandatory dataset in collaboration with participating sites to enable increased data completeness and improve data quality.
- Consider introducing a uniform template which is consistent between data submissions to harmonize the order and coding of variables.
- Further reduce the substantial inter- and intra-country differences in surveillance methods to ensure homogeneity of the scope, focus, objectives, methodology, resources and reporting across different regions and countries.

#### Communication

- Continue further data analysis to identify preventive and risk factors with the available data collected through the surveillance and share results with the network and scientific community.

#### Sustainability

- Monitor outbreak-related workload issues to ensure the sustainability of the surveillance.
- Consider lessons learned from the I-MOVE-COVID-19 hospital surveillance network when data submission continues under other networks (VEBIS) to ensure sustainability.

## Conclusion

This evaluation found that the COVID-19 hospital surveillance system met its surveillance objectives by describing COVID-19 cases with severe disease and outcomes. There were clear-cut added benefits for stakeholders from being involved in a collaborative European network. The COVID-19 pandemic, an unprecedented event, put an immense pressure on respiratory surveillance systems. The proposed recommendations presented here identify learning opportunities to support preparedness and surveillance response for future pandemics. Finally, the evaluation framework applied in this paper can be instrumental for other European surveillance system evaluations, particularly for those established as a result of the COVID-19 pandemic.

## Data Availability

The data underlying this article can be shared upon reasonable request to the corresponding author. Key points This rapidly deployed European surveillance network was an important enhancement to existing surveillance mechanisms used in Europe and participation in this collaborative European network was considered beneficial for stakeholders. The surveillance system could easily be adapted to include additional respiratory pathogens and be used by countries with less surveillance capability to make them resilient and agile for addressing global and national respiratory surveillance needs. The lessons we learned during this evaluation are critical to the successful implementation of novel long-term respiratory surveillance projects being carried out across Europe in the aftermath of the COVID-19 pandemic.
